# Osteogenic Effect and Cell Signaling Activation of Extremely Low-Frequency Pulsed Electromagnetic Fields in Adipose-Derived Mesenchymal Stromal Cells

**DOI:** 10.1155/2018/5402853

**Published:** 2018-07-12

**Authors:** Patrina S. P. Poh, Claudine Seeliger, Marina Unger, Karsten Falldorf, Elizabeth R. Balmayor, Martijn van Griensven

**Affiliations:** ^1^Experimental Trauma Surgery, Department of Trauma Surgery, Klinikum Rechts der Isar, Technical University of Munich, Ismaninger Strasse 22, 81675 Munich, Germany; ^2^Sachtleben GmbH, Hamburg, Germany

## Abstract

Extremely low-frequency pulsed electromagnetic field (ELF-PEMF) devices have been used in the clinic for the treatment of bone disorders over the past 30 years. However, the underlying mechanism of which ELF-PEMFs exert an effect on tissues at a cellular level is not well understood. Hence, in this study, we explored the potential of different ELF-PEMF signals in modulating human adipose-derived mesenchymal stromal cells' (hAMSC) osteogenic capability. The cell proliferation rate was assessed using carboxyfluorescein succinimidyl ester (CFSE) method. The osteogenesis potential of cells was determined by alkaline phosphatase (ALP) activity, Alizarin-Red S staining, and RT-qPCR. Finally, the intracellular signaling pathway of a selected ELF-PEMF signal was examined using the PathScan Intracellular Signaling Array. Among the tested ELF-PEMF signals, program 20 (26 Hz) showed activation of the Akt and MAPK/ERK signaling cascade and significant upregulations of collagen I, alkaline phosphatase, and osteocalcin when compared to nonstimulated cells. This study demonstrates the potential of certain ELF-PEMF signal parameters to induce osteogenic differentiation of hAMSC and provides important clues in terms of the molecular mechanisms for the stimulation of osteogenic effects by ELF-PEMF on hAMSC.

## 1. Introduction

Clinical intervention of large bone defects is limited. Autografts (transplantation of patient's own tissue) remain the gold standard for treating large bone defects. Despite exhibiting high healing rates, autografts have associated disadvantages; approximately 20–30% of autograft patients experienced donor site morbidity and are complicated by fracture, nonunion, and infection. Therefore, effective treatments for such bone defects are urgently needed.

Over the years, cell therapy has been proven to be a viable strategy that can aid the process of bone regeneration [1]. Autologous adipose-derived mesenchymal stromal cells (AMSC) are a promising tool in cell therapy due to their relative ease to harvest compared to other sources of mesenchymal stromal cells (MSC) and have been indicated as a cell source with high regenerative potential [1, 2]. However, the efficacy of AMSC therapy depends upon how effectively transplanted AMSC can be targeted persistently to the diseased area and how functional these cells are in terms of the regeneration process. Bone regeneration is a very dynamic and complex process involving diversity of cell types whose functions are regulated by intricate networks of biochemical signals. One crucial phase of bone regeneration is the proliferation and differentiation of precursor cells (i.e., MSC) into osteoblasts (bone-forming cells) that would build up the mineralized bone matrix. Hence, there have been tremendous efforts in the development of noninvasive strategies, which could complement cell therapy by stimulating proliferation and guiding differentiation of MSC within the injured sites to promote bone regeneration [3, 4]. Among these, ELF-PEMFs present a potential technology platform, which can be applied noninvasively to regulate desirable cellular responses. ELF-PEMF-generating devices can produce electromagnetic signals with specific amplitudes, frequencies, and waveforms [5]. These signals can be transduced into soft tissue through an external coil applied at the intended injury sites, resulting in localized induced electric and magnetic fields [6]. Some studies suggested improved bone regenerative capabilities favoring osteoblast proliferation, differentiation, and production of calcified extracellular matrix (ECM) as a result of exposures to ELF-PEMF signals [7–12].

ELF-PEMF therapies aimed at aiding fracture repair have been investigated clinically for more than 30 years. Many efforts have been geared towards understanding the fundamental mechanism of ELF-PEMF stimulation on MSC harvested from different sources (i.e., alveolar bone-derived MSC [13], bone marrow-derived MSC (BMSC), and AMSC [14, 15]) and the associated implications on bone regeneration. However, while promising results have been obtained, there is still no clarity on the nature of such mechanism of action or on the optimal ELF-PEMF signal parameters which can be utilized to enhance osteogenic capabilities. Because of this, the optimal ELF-PEMF signal configurations required to enhance osteogenic potential of hAMSC [14–17] are uncertain. In most studies, the amplitude and frequency of the ELF-PEMF signal used to induce osteogenesis varied from 0.1 to 3 mT and from 7.5 to 75 Hz, respectively [4, 16], showing varying outcomes depending on the ELF-PEMF configurations (i.e., frequency, amplitude, and waveforms), ELF-PEMF devices (i.e., shape and size of applicator/field coil), method of application (i.e., position of the applicator in respect to the cells'/tissues' position), and duration of exposure. In this regard, for example, exposure durations found in the literature vary from 5 mins to 14 hours per day [5, 18] with no consensus on the optimal treatment duration. However, at present, long-term exposure of organs and tissues to ELF-PEMF is still highly debatable [19]. *In vivo* studies have illustrated that long-term exposure to ELF-PEMF can cause negative side effects, such as reduced sperm motility and testosterone level (1 mT, 50 Hz EMF, 24 hrs for 85 days) [20] and enhanced oxidative stress in liver tissue (1 mT, 50 Hz EMF, 4 hrs per day for 45 days) [21]. On the other hand, short exposures have shown promising benefits in line with those expected from potential therapies [22].

Within this context, we performed this study in an attempt to identify further potential ELF-PEMF signals that can potentially guide or enhance the osteogenic capabilities of hAMSC. Subsequently, the intracellular signaling pathways activated in hAMSC due to exposure of ELF-PEMF were examined.

## 2. Materials and Methods

### 2.1. Isolation of hAMSC

Isolation of hAMSC from 6 donors (*N* = 6) was performed with written informed patient's consent (acquired prior to tissue collection) and with approval of the local ethical committee of the University Hospital “Klinikum Rechts der Isar”, Technical University of Munich, Germany, and according to the ethical guidelines established by this institution as well as the Declaration of Helsinki in its latest amendment. Briefly, solid fat samples (manually minced into smaller pieces) or liposuctions were digested with 0.075% (*w*/*v*) collagenase type II (Biochrom, Germany) in PBS at 37°C for 30 min. The digestion was terminated using DMEM-high glucose supplemented with 100 U/ml penicillin, 100 *μ*g/ml streptomycin, and 10% (*v*/*v*) fetal calf serum (FCS). Detailed cell isolation procedures have been described by Schneider et al. [23]. The isolated cells were incubated at 37°C, 5% CO_2_, and 95% air humidity. Cells of passage 3 were used for cell proliferation and mineralization assays, while gene expression and intracellular signaling arrays were performed with cells of passage 4.

### 2.2. ELF-PEMF Exposure

**Figure 1:**
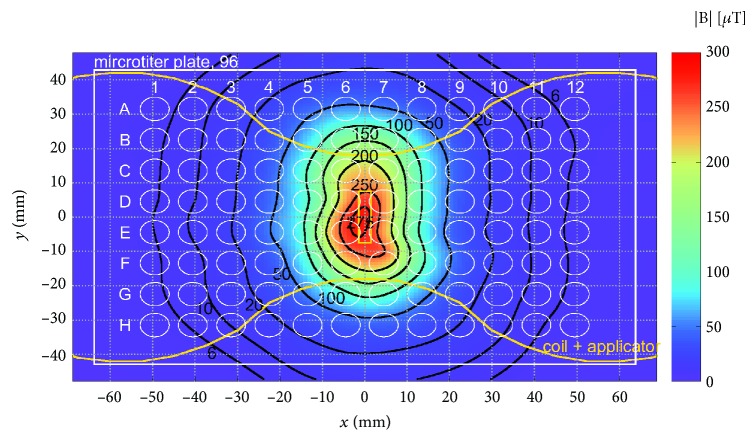
Peak magnetic field magnitude distribution across the cell culture plate (6 mm above the applicator). Yellow line outlined the contour of the applicator.

To evaluate the proliferative and osteogenic differentiation behavior of hAMSC when exposed to different ELF-PEMF signals, cell culture plates/flasks were placed onto the applicator (Figure 1). The applicator was connected to the Somagen® device (CE 0482, compliant with EN ISO 13485:2012 + AC:2012, Sachtleben GmbH, Germany) where ELF-PEMF signals were generated as previously described [24–26]. For the present study, 10 different ELF-PEMF signals (termed “CIT programs” by the manufacturer) were used. Briefly, all the ELF-PEMF signals generated by the device were constituted by a fundamental pulse, which was arranged into a pulse train with different fundamental frequencies as listed in Table 1.

Figure 1 shows the distribution of the peak ELF-PEMF magnetic field magnitude as a measure of the field homogeneity over the cell culture plate.

### 2.3. Cell Culture

The cell culture studies were divided into two sequential parts. In the first part (study 1), hAMSCs were trypsinised and plated with a density of 1 × 10^4^ cells/cm^2^ in 1 96-well plate. The osteogenic differentiation of hAMSC was induced using DMEM-low glucose supplemented with 100 U/ml penicillin, 100 *μ*g/ml streptomycin, 5% (*v*/*v*) FCS, 100 nM dexamethasone, 1.6 mM calcium chloride (CaCl_2_), 25 mM HEPES, 0.2 mM ascorbic acid, and 10 mM *β*-glycerol phosphate (hereafter referred as osteogenic medium). Then, cells were separately treated with each one of the ELF-PEMF signals listed in Table 1, once a day for 7 mins over 2 weeks. At days 3, 7, and 14, immediately after the ELF-PEMF exposure, cells were harvested for cell proliferation and mineralization assays.

In the second part (study 2), hAMSCs were identically plated at a density of 1 × 10^4^ cells/cm^2^ and cultured in osteogenic medium. The cells were exposed to one ELF-PEMF signal identified in study 1 also for 7 mins (single exposure). After a defined resting period (of 30 mins, 1, 2, 3, 4, and 6 hrs), samples were collected for intracellular cell signaling arrays. In both studies, hAMSCs cultured in osteogenic medium without ELF-PEMF exposure were used as control.

### 2.4. Carboxyfluorescein Succinimidyl Ester (CFSE) Labelling of Cells

Cell proliferation was assessed using CFSE (Abcam®, ab113853, UK). Briefly, cells were fluorescence labelled with 1 *μ*M CFSE in culture media for 15 mins at 37°C. Then, the cells were washed with culture media to remove nonincorporated dye and topped up with fresh culture media. On days 3, 7, and 14, cells were harvested and CFSE fluorescence absorbance was detected using a flow cytometer (MACSQuant, Miltenyi Biotec, Germany) using the blue laser (488 nm) and a 525/50 nm filter.

### 2.5. Sulforhodamine B (SRB) Staining of Cellular Protein

At days 3, 7, and 14, cells were fixed with ice-cold methanol for 15 mins and incubated in SRB solution for 30 mins. Subsequently, cells were washed with 1% acetic acid solution to remove unbound SRB. Then, SRB was incubated with 10 mM unbuffered Tris solution for 15 mins to dissolve bound SRB. Finally, absorbance was measured at 565/690 nm using a plate reader (BMG Labtech, Germany). A standard curve with known protein amount was generated and used for the calculation of corresponding value for all samples.

### 2.6. Alkaline Phosphatase (ALP) Activity

On days 3, 7, and 14, cell culture medium was aspirated, and cells were washed once with PBS. Then, cells were covered with ALP substrate solution (i.e., 3.5 mM 4-disodium-4-nitrophenyl phosphate prepared in 0.1 M AP-buffer consisting of 50 mM glycine, 100 mM Tris-base, and 2 mM magnesium chloride at pH 10.5) for 30 min at 37°C. Subsequently, 100 *μ*l of reaction mixture was transferred into a 96-well plate in triplicate. Absorption of the reaction product was measured at 405 nm using a plate reader (BMG Labtech, Germany). Presented data were normalized to protein content.

### 2.7. Von Kossa Staining for Matrix Mineralization

At different time points (3, 7, and 14 days), the culture was washed twice with PBS and fixed with ice-cold methanol for 15 mins. Subsequently, culture was stained with 3% (*w*/*v*) silver nitrate solution (Fisher, Germany) for 30 mins, followed by three rinses with dH_2_O. Stain was developed in 1% (*w*/*v*) pyrogallol solution for 3 mins, followed by two rinses with dH_2_O (5 mins each). Then, culture was incubated with 5% sodium thiosulfate solution for 5 mins, washed in running tap water for 15 mins, and incubated with Kernecht-red solution for 5 mins. After washing, culture was treated with 96% ethanol for 1 min, air dried, and imaged using light microscopy (BZ-9000, Keyence, Japan).

### 2.8. Alizarin Red S Staining for Semiquantification of Matrix Mineralization

This procedure was performed for semiquantification of the extent of matrix mineralization. Briefly, on days 3, 7, and 14, cells were washed with PBS and fixed with ice-cold ethanol for 30 mins at room temperature. Then, cells were washed with distilled water (dH_2_O) and incubated with 0.5% (*w*/*v*) Alizarin Red S in dH_2_O, pH 4 for 10 min. After that, unincorporated dye was washed away using several rinses of dH_2_O. The precipitates were dissolved using 10% hexadecylpyridinium chloride, and 100 *μ*l of the solution was transferred to a 96-well plate in triplicate. The absorption of the reaction product was measured at 562 nm using a plate reader. Presented data were normalized to protein content.

### 2.9. RT-qPCR

On days 3, 7, and 14, the cells were harvested, and mRNA was extracted using Tri Reagent (Sigma-Aldrich, Germany) according to manufacturer's recommendations. RNA quantification and quality control were done with NanoDrop (Nanodrop Tech, USA). Reverse transcription to cDNA was performed in a C1000 Touch Thermal Cycler (Eppendorf, Germany) using a first strand cDNA synthesis kit (Thermo Scientific, USA) and following the instructions of the manufacturer. qPCR was performed in a CFX96 Real-Time System thermocycler using SsoFast EvaGreen supermix (Bio-Rad, Hercules, CA, USA) as a detection reagent for the gene expression of RunX2, osteocalcin, osteopontin, osterix, ALP, and collagen type I (COLIAI). Primer sequences used are listed in Table 2. Gene expression is expressed as 2^−ddCT^ relative to the housekeeper (*β*-tubulin) and to the control group.

### 2.10. Intracellular Cell Signaling Array

The intracellular cell signaling pathway was examined using the PathScan Intracellular Signaling Array Kit (Cell Signaling Technology, The Netherlands) following the manufacturer's protocol. Briefly, cells were lysed using ice-cold lysis buffer and the lysates were diluted to 1 mg protein per ml solution using array diluent buffer. Following that, 75 *μ*l of lysate was added to nitrocellulose-coated glass slides precoated with primary antibodies. The plate was incubated overnight at 4°C. Following washing using the array wash buffer, 75 *μ*l of detection antibody cocktail was added to each well and incubated for an hour at room temperature on an orbital shaker. Following washing steps, 75 *μ*l of HRP-linked streptavidin was added to each well and incubated at room temperature for 30 mins on an orbital shaker. Lastly, 1x LumiGLO®/peroxide reagent was added and chemiluminescence was detected using a chemiluminescence imager (Bio-Rad, USA).

### 2.11. Statistical Analysis

Cell proliferation, protein content, ALP activity, and Alizarin Red S data were represented as mean ± standard error of mean (SEM). Cell signaling protein array data was represented as mean ± standard deviation (SD). The cell proliferation, protein content, ALP activity, and Alizarin Red S data were subjected to two-way analysis of variance (two-way ANOVA) and Tukey's post hoc test (GraphPad Prism 7.0). Significance level was set at *p* < 0.05. On the other hand, RT-qPCR and cell signaling array data were subjected to one-way analysis of variance (one-way ANOVA) and Student's unpaired two-tailed *t*-test.

## 3. Results

### 3.1. Impact of ELF-PEMF Exposure on hAMSC Proliferation

**Figure 2:**
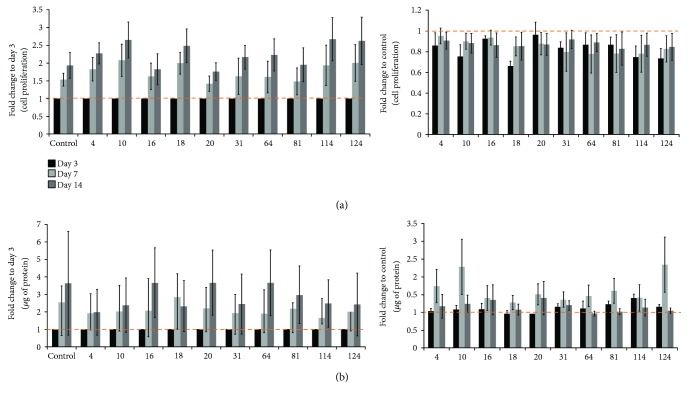
Graphs show fold change of (a) cell proliferation and (b) protein content to the respective day 3 of the group (left graphs) and to the control group (without ELF-PEMF exposure) of the same culture period (e.g., 3 versus 3 days, 7 versus 7 days, and 14 versus 14 days) (right graphs). Mean ± SEM, *N* = 6 donors, *n* = 3 triplicates.

Cell proliferation was assessed by CFSE and SRB (protein content). CFSE is a membrane-permeant fluorescent dye that covalently attaches to free amines of cytoplasmic proteins, and the CFSE fluorescence within daughter cells progressively halved following each cell division [27]. On the other hand, SRB binds stoichiometrically to cellular proteins and can be easily eluted and used as a proxy for cell proliferation [28]. In all groups, cell proliferation increased steadily from day 3 to day 14. However, when compared to the control group of the same culture period, cell proliferation slightly decreased after 3 days of ELF-PEMF exposure. However, no significant differences on cell proliferation were detected between groups (Figures 2(a) and 2(b)).

### 3.2. Osteogenic Capabilities of hAMSC When Treated with ELF-PEMF

**Figure 3:**
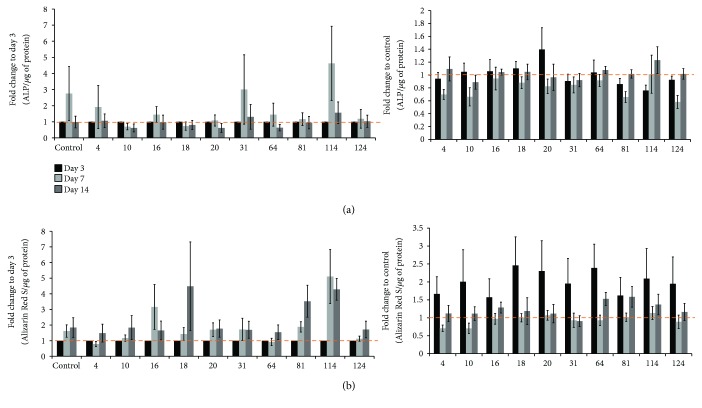
Graph shows fold change of (a) alkaline phosphatase (ALP) and (b) Alizarin Red S content to the respective day 3 cultured group and to the control (without ELF-PEMF exposure) of the same culture period (e.g., 3 versus 3 days, 7 versus 7 days, and 14 versus 14 days). Mean ± SEM, *N* = 6 donors, *n* = 3 triplicates.

All cells were cultured under osteogenic media to determine if the application of ELF-PEMF would enhance the differentiation of hAMSC towards osteogenic lineage. ALP activity slightly increased for control, ELF-PEMF groups 4, 31, and 114 at day 7 compared to the respective day 3 of the group (Figure 3(a)). However, only CIT number 20 exposed group showed slight increase in ALP activity compared to control on day 3 (Figure 3(a)).

**Figure 4:**
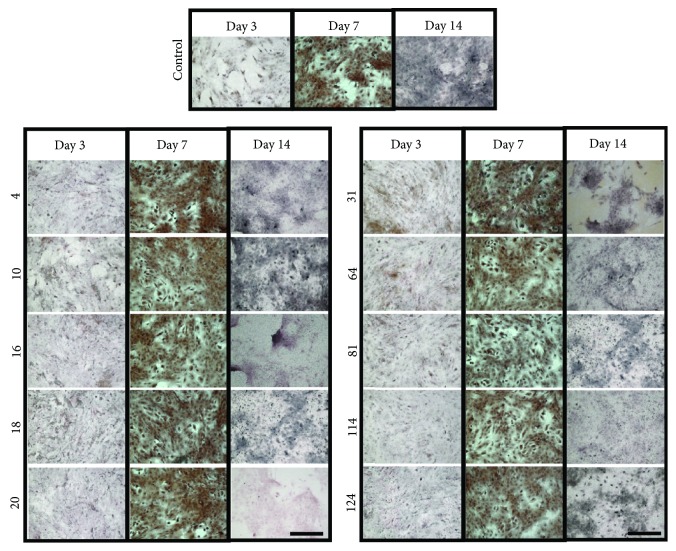
Representative images of culture stained with von Kossa on days 3, 7, and 14 immediately after exposure with ELF-PEMF. Control: cells without ELF-PEMF exposure. Scale bar = 500 *μ*m.

Generally, Alizarin Red S and von Kossa assays (Figures 3(b) and 4) showed that increasing the amount of mineralized matrix was formed over time in all groups, with or without ELF-PEMF exposure. It was noted that the effect of ELF-PEMF on matrix mineralization was more prominent on day 3, as all ELF-PEMF-treated groups showed 1.5- to 2.5-fold increase in Alizarin Red S staining compared to control (Figure 3(b)).

**Figure 5:**
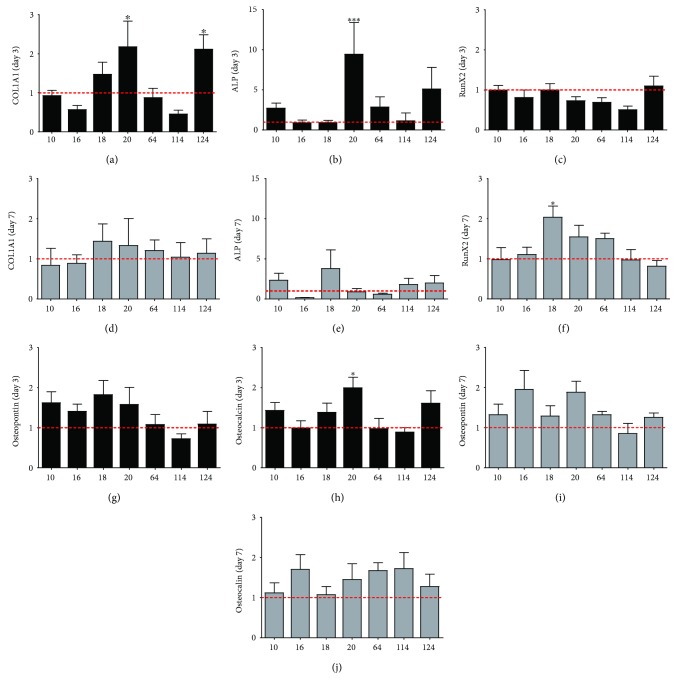
Graph showing the relative gene expression of (a, d) COLIAI, (b, e) alkaline phosphatase (ALP), (c, f) RunX2, (g, i) osteopontin, and (h, j) osteocalcin after (a–c, g, h) 3 and (d–f, i, j) 7 days in culture under the various ELF-PEMF signals. Data were plotted as fold change to control of the same culture period, represented by the dotted line (*y* = 1). Mean ± SEM, *N* = 5 donors, *n* = 3 triplicates (^∗^*p* < 0.05 and ^∗∗∗^*p* < 0.001).

Of the ten different ELF-PEMF signals, seven (e.g., CIT numbers 10, 16, 18, 20, 64, 114, and 124) were further investigated in terms of osteogenic gene expression (Figure 5).

Cells treated with CIT numbers 20 and 124 showed significant upregulation of COLIAI after 3 days of exposure compared to control. Moreover, CIT number 20 also resulted in a significant upregulation of ALP and osteocalcin for the same time of observation. On the other hand, CIT number 18-treated cells showed significant upregulation of RunX2 expression after 7 days compared to control. Osteopontin expression levels were slightly, but not significantly, elevated for all ELF-PEMF groups except for CIT number 114 on both days 3 and 7 compared to control.

### 3.3. Effect of 26 Hz ELF-PEMF on Intracellular Cell Signaling of hAMSC

**Figure 6:**
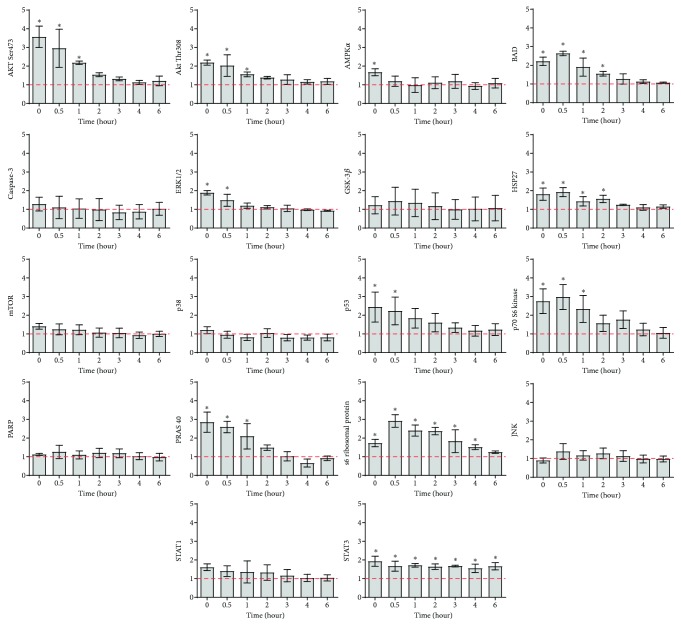
Graph shows the levels of key phosphorylated/cleaved proteins involved in the Akt, MAPK/ERK, and caspase signaling pathways in hAMSC subjected to 26 Hz ELF-PEMF (CIT number 20) exposure in osteogenic media. Protein quantification was examined immediately, 0.5, 1, 2, 3, 4, or 6 hours after the completion of the ELF-PEMF exposure. Data were plotted as fold change to control (hAMSC culture in osteogenic media without ELF-PEMF exposure), represented by the line (*y* = 1). Mean ± SD, *N* = 2 donors, *n* = 4 replicates. ^∗^*p* < 0.05 compared to control.

Based on the observations from part 1 of the study, it was indicated that 26 Hz ELF-PEMF (CIT number 20) shows potential of triggering osteogenesis of hAMSC (upregulation of COL1A1, ALP, and osteocalcin genes at day 3) as compared to other ELF-PEMF signals. Thus, this signal was selected for the elucidation of intracellular cell signaling pathways using a protein array. The system used allowed for the detection of 18 different proteins involved in proliferation, growth, apoptosis, and/or stress signaling when phosphorylated or cleaved.  Figure 6 shows the state of various intracellular proteins measured in hAMSC treated with CIT number 20 for 7 mins. Measurements were performed either immediately (without resting period) or after 0.5, 1, 2, 3, 4, and 6 hours of resting period.

Generally, it was observed that the amount of phosphorylated Akt, AMPK*α*, BAD, ERK1/2, HSP27, p53, p70 S6 kinase, PRAS 40, and s6 ribosomal protein increased during exposure and gradually returned to baseline as hAMSCs were left at resting state (Figure 6). Conversely, cleavage of caspase-3 and PARP as well as phosphorylation of GSK 3*β*, mammalian target of rapamycin (mTOR), p38, JNK, and STAT1 did not change significantly throughout the experimental period (Figure 6). Notably, the amount of phosphorylated STAT3 significantly increased compared to the control sample and was kept almost constant for up to 6 hours after the completion of the exposure (Figure 6).

## 4. Discussion

Across all tested ELF-PEMF programs, none caused irreversible cytotoxicity to hAMSC as evidenced by the increasing cell proliferation and protein production over time. This observation was in line with that of intracellular signaling array. When exposed to 26 Hz ELF-PEMF (CIT number 20), a stress-related pathway was activated. This is evidenced by the increase in HSP27 activation, which has been shown to protect cells from undergoing apoptosis under stress conditions [29] through interactions with cytochrome-c or caspase-3 [30]. Additionally, p53, an antioncogenic protein, which plays an important role in response to DNA damage [31], was also activated. Notably, apoptosis was repressed, as shown by the activation of the prosurvival Akt pathway due to the lack of activation of the proapoptotic effectors caspase-3 [32] and PARP and the inactivation of the proapoptotic protein BAD.

In this study, ELF-PEMFs were applied to hAMSC cultured in osteogenic-conditioned media to elucidate the potential of ELF-PEMF in acceleration of hAMSC osteogenesis differentiation. All groups (including control) show a general increase of mineralized matrix over time. However, only prominent differences in terms of extent of mineralization were observed on day 3 in ELF-PEMF-treated groups compared to control. This indicated that ELF-PEMFs do elicit a positive response towards hAMSC matrix mineralization. However, in our experiments, we noted a dramatic difference in terms of the capability to form mineralized matrix among the different donor hAMSC as reflected in the relatively large deviation (Figure 3) and von Kossa staining of different donor hAMSC with and without ELF-PEMF exposures (Figure 4).

At the gene level, it was observed that hAMSC exposed to 26 Hz ELF-PEMF (CIT number 20) exhibited a more prominent differentiation into osteogenic lineage compared to other exposure groups as evidenced by the increase in ALP (an early marker for osteoblast progenitors), COLIAI (collagen type I, the most abundant ECM protein in bone tissue), and osteocalcin (secreted by mature osteoblasts and commonly used to represent terminal osteoblast differentiation) gene expression after 3 days of exposure. The temporal expression of osteogenic-related genes can be utilized to characterize osteoblasts' maturation process [33]. This indicated that CIT program number 20 is relatively more effective when compared to other tested ELF-PEMF programs in inducing hAMSC towards the osteogenic lineage.

Based on the gene expression profiles, a protein array was chosen to further elucidate the underlying intercellular signaling pathways that lead to enhanced hAMSC potential for osteogenic differentiation. Akt signaling cascade was activated in hAMSC when exposed to 26 Hz ELF-PEMF (CIT number 20), marked by the increased levels of phosphorylated Akt at Ser473 and Thr308, p70 S6 kinase, and S6 ribosomal protein [34]. On the other hand, there were no significant changes in the levels of phosphorylated mTOR after exposure. However, it is well established that mTOR regulates protein synthesis through the phosphorylation and activation of S6 kinase, which is used as a readout of mTOR activity [34].

**Figure 7:**
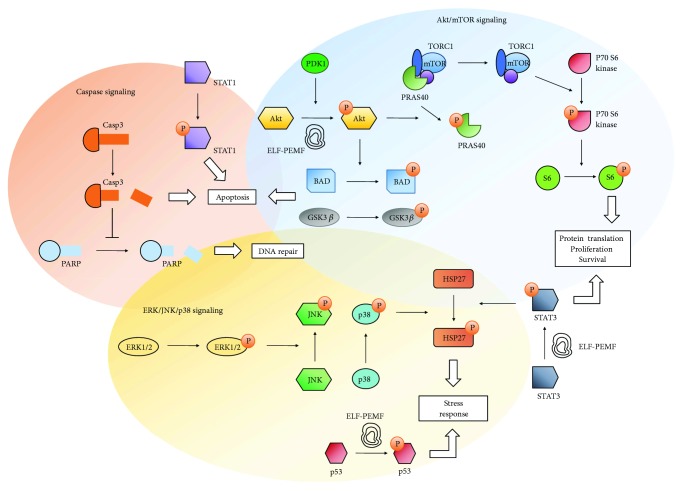
Illustration of the intracellular signaling pathways investigated on human adipose-derived mesenchymal stromal cells when subjected to ELF-PEMF. The coactivation of ERK and Akt in response to CIT program number 20 exposure promoted cell growth and survival and prevented apoptosis despite of certain levels of cellular stress.

Our results also suggest coactivation of ERK 1/2 signaling pathway in hAMSC exposed to 26 Hz ELF-PEMF, as shown by the increased levels of phosphorylated ERK1/2 shortly after exposure. Studies have reported that the Akt [35] and ERK1/2 [36] signaling pathways can be activated during cell stress to prevent cell death and work synergistically to promote cell proliferation and protein translation [37]. This happens because Akt activation promotes nuclear export of p21cip1 into the cytoplasm, where it is degraded by the proteasome. As a result, p21cip1 levels decrease and the cell cycle arrest ceases [38]. These observations suggest that a combination of ERK and Akt activation (Figure 7) in response to 26 Hz ELF-PEMF promoted hAMSC growth and survival and prevented apoptosis despite of certain levels of cellular stress. This hypothesis is supported by the observed long-lasting phosphorylation of STAT3, a crucial transcription factor implicated in the maintenance and antiapoptotic status of cells [22]. It is noted that further experiments are required to confirm these initial observations.

Recently, using the same ELF-PEMF device, Ehnert et al. [24] demonstrated that primary human osteoblasts subjected to 16 Hz ELF-PEMF (CIT number 16) exposure showed better viability and maturation through the activation of the ERK1/2 signaling cascade. On the contrary, it did not affect osteoclast viability and maturation. Furthermore, antioxidative defense mechanisms could be induced by the same ELF-PEMF signal as reported by Ehnert et al. [24]. More recently, Ehnert et al. [26] also showed the potential of ELF-PEMF 16 Hz (CIT number 16) and 26 Hz (CIT number 20) in promoting osteogenesis in coculture of osteoblasts and MSCs and an increase in osteoclast activity when exposed to 26 Hz ELF-PEMF (CIT number 20). Collectively, these data indicate how cells of different lineages respond differently to ELF-PEMF signals. Future studies will be performed on a more complex culture system (e.g., co-/triculture model incorporating osteoblasts, osteoclasts, and monocytes/macrophages) to elucidate a more effective ELF-PEMF signal and spatial configuration, which can exert a net positive bone formation response.

## Figures and Tables

**Table 1:** ELF-PEMF signals utilized in the study. Frequencies (pulse repetition rate) of the ELF-PEMF signals investigated.

“CIT program number”	Pulse repetition rates (Hz)
4	10.0
16	16.0
10	20.6
18	23.8
20	26.0
64	33.0
31	49.9
81	52.3
114	75.6
124	90.6

**Table 2:** Forward and reverse primer sequences and annealing temperature for the respective genes.

Gene	Forward primer	Reverse primer	Annealing temperature (°C)
RunX2	TGCCTAGGCGCATTTCAGGTGC	TGAGGTGATGGCGGGGTGT	60
Osteocalcin	CCAGCGGTGCAGAGTCCAGC	GACACCCTAGACCGGGCCGT	60
Osteopontin	CTCCATTGACTCGAACGACTC	CGTCTGTAGCATCAGGGTACTG	60
Alkaline phosphatase	ACGTGGCTAAGAATGTCATC	CTGGTAGGCGATGTCCTTA	57.5
Collagen I	AGCGGACGCTAACCCCCTCC	CAGACGGGACAGCACTCGCC	60
Βeta-tubulin	GAGGGCGAGGACGAGGCTTA	TCTAACAGAGGCAAAACTGAGCACC	60

## Data Availability

The quantitative data from, that is, ALP, RT-qPCR, and intracellular signaling pathway array used to support the findings of this study are available from the corresponding author upon reasonable request. The ELF-PEMF signal pattern data used to support the findings of this study were supplied by Sachtleben GmbH, Hamburg, Germany, and so cannot be made freely available. Requests for access to these data should be made to Karsten Falldorf at falldorf@citresearch.de.
